# Comparison of chemical stability and corrosion resistance of group IV metal oxide films formed by thermal and plasma-enhanced atomic layer deposition

**DOI:** 10.1038/s41598-019-47049-z

**Published:** 2019-07-18

**Authors:** Min Li, Zhi-Xian Jin, Wei Zhang, Yu-Hang Bai, Yan-Qiang Cao, Wei-Ming Li, Di Wu, Ai-Dong Li

**Affiliations:** National Laboratory of Solid State Microstructures, Department of Materials Science and Engineering, College of Engineering and Applied Sciences, Collaborative Innovation Center of Advanced Microstructures, Nanjing University, Nanjing, 210093 People’s Republic of China

**Keywords:** Corrosion, Chemical physics

## Abstract

The wide applications of ultrathin group IV metal oxide films (TiO_2_, ZrO_2_ and HfO_2_) probably expose materials to potentially reactive etchants and solvents, appealing for extraordinary chemical stability and corrosion resistance property. In this paper, TiO_2_ ultrathin films were deposited on Si at 200 °C while ZrO_2_ and HfO_2_ were grown at 250 °C to fit their growth temperature window, by thermal atomic layer deposition (TALD) and plasma-enhanced ALD (PEALD). A variety of chemical liquid media including 1 mol/L H_2_SO_4_, 1 mol/L HCl, 1 mol/L KOH, 1 mol/L KCl, and 18 MΩ deionized water were used to test and compare chemical stability of all these as-deposited group IV metal oxides thin films, as well as post-annealed samples at various temperatures. Among these metal oxides, TALD/PEALD HfO_2_ ultrathin films exhibit the best chemical stability and anti-corrosion property without any change in thickness after long time immersion into acidic, alkaline and neutral solutions. As-deposited TALD ZrO_2_ ultrathin films have slow etch rate of 1.06 nm/day in 1 mol/L HCl, however other PEALD ZrO_2_ ultrathin films and annealed TALD ones show better anti-acid stability, indicating the role of introduction of plasma O_2_ in PEALD and post-thermal treatment. As-deposited TiO_2_ ultrathin films by TALD and PEALD are found to be etched slowly in acidic solutions, but the PEALD can decrease the etching rate of TiO_2_ by ~41%. After post-annealing, TiO_2_ ultrathin films have satisfactory corrosion resistance, which is ascribed to the crystallization transition from amorphous to anatase phase and the formation of 5% Si-doped TiO_2_ ultrathin layers on sample surfaces, i.e. Ti-silicate. ZrO_2_, and TiO_2_ ultrathin films show excellent corrosion endurance property in basic and neutral solutions. Simultaneously, 304 stainless steel coated with PEALD-HfO_2_ is found to have a lower corrosion rate than that with TALD-HfO_2_ by means of electrochemical measurement. The pre-treatment of plasma H_2_ to 304 stainless steel can effectively reduce interfacial impurities and porosity of overlayers with significantly enhanced corrosion endurance. Above all, the chemical stability and anti-corrosion properties of IV group metal oxide coatings can be improved by using PEALD technique, post-annealing process and plasma H_2_ pre-treatment to substrates.

## Introduction

Group IV metal oxide films deposited via atomic layer deposition (ALD) including titania (TiO_2_), zirconia (ZrO_2_) and hafnia (HfO_2_) have been widely investigated due to their excellent properties in electrical, optical, photocatalytical, biological and mechanical fields^1–6^. During certain utilization process, these films have to work in harsh environments such as chemical liquid media of acid and alkaline with a wide range of pH value to maintain their desired properties. ALD is a competitive technique for thin film deposition based on sequential self-limited reaction mechanism using precursor vapor, which has many unique advantages such as large area uniformity, excellent three-dimensional conformality, simple and precise control in film thickness, dense and pinhole-free films, and low processing temperature^7,8^. Compared with the conventional thermal ALD (TALD), plasma-enhanced ALD (PEALD) is an energy-assisted method for fabrication of thin films, where plasma species are utilized as reactive gas during one step of the cyclic deposition process^9^. It can produce some virtues over the TALD route, including more choice of materials and precursors, substrate temperature at room temperature, improved film density and quality with high purity and little defects, etc.^10^. Therefore, extensive and intensive studies on ALD-derived ultrathin films as protective coatings and anti-corrosion barriers are needed greatly. In consideration of better film quality, PEALD may be the more suitable candidate to obtain the ultrathin corrosion-resistant coatings with long-term stability in severe environments^11,12^.

So far, some researches on surface passivation and chemical protection by thermal ALD-derived thin films have been performed^13^, the majority of which focus on several common metal oxides like alumina and titania formed by TALD and their stability in various environments, especially in aqueous solutions for photoelectrochemical water splitting^14^ and protection of stainless steel^15,16^ or other metal substrates^17,18^. For example, Abdulagatov *et al*. deposited Al_2_O_3_ and TiO_2_ on Cu and found that only Al_2_O_3_ or TiO_2_ TALD films were insufficient to prevent copper from corrosion whereas the introduction of TiO_2_ capping layer on Al_2_O_3_ films was more resilient to dissolution in water^18^. Strandwitz *et al*. have demonstrated TALD-derived MnO thin films can be used to stabilize n-Si photoelectrodes in chemically reactive conditions^19^. The chemical stability of TALD Al_2_O_3_ and TiO_2_ films in different acidic, basic and neutral media, and the influence of post-deposition thermal treatment on different samples have been discussed^20^. These valuable results are helpful to expansive applications of organic electronic devices^21^, stabilization of semiconductor photoanodes for water oxidation^22^ and many other fields. However, to date, the work on PEALD metal oxide thin films as protective layers and anti-corrosion barriers is rather lacking^11,12,21^.

In this paper, the chemical stability of as-deposited and post-annealed TiO_2_, ZrO_2_ and HfO_2_ thin films derived from TALD and PEALD in various chemical liquid media, including 1 mol/L H_2_SO_4_, 1 mol/L HCl, 1 mol/L KOH, 1 mol/L KCl, and pure water, has been investigated and compared systematically. A series of analytical methods were used to characterize the change of thickness, chemical composition, structure and morphology of different samples. It is found that TALD/PEALD HfO_2_ films exhibit the best chemical stability and anti-corrosion property without any change in thickness after long time immersion into various chemical solutions. The corrosion endurance of HfO_2_ coating for 304 stainless steel (SS) in 1 mol/L KCl solution has been studied by *in-situ* electrochemical characterization.

## Results and Discussion

### Composition, morphology and structure

**Figure 1:**
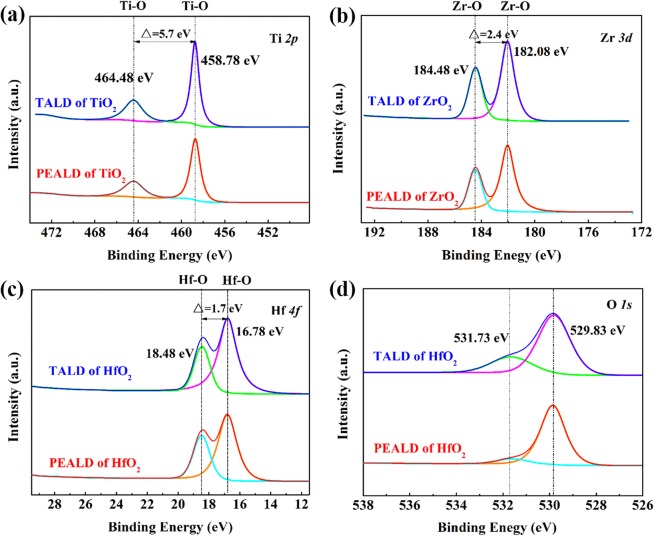
Narrow-scan Ti *2p*, Zr *3d* and Hf *4f* XPS spectra of as-deposited TALD and PEALD (**a**) TiO_2_, (**b**) ZrO_2_ and (**c**) HfO_2_ films on Si. (**d**) Typical O *1s* XPS spectra of as-deposited TALD and PEALD HfO_2_ films on Si.

Chemical composition of all as-deposited TALD and PEALD samples on Si was examined by XPS, as seen in Fig. 1. The strong doublet peaks of Ti *2p* (a), Zr *3d* (b) and Hf *4f* (c) with respective splitting energy of 5.7 eV, 2.4 eV, and 1.7 eV, and the strong O *1s* signal at 529.83 eV (d, only showing TALD and PEALD HfO_2_) verify the formation of TiO_2_, ZrO_2_ and HfO_2_ films by TALD and PEALD. Herein the weak O *1s* peak at 531.73 eV might come from the surface adsorption oxygen of HfO_2_ films.

**Figure 2:**
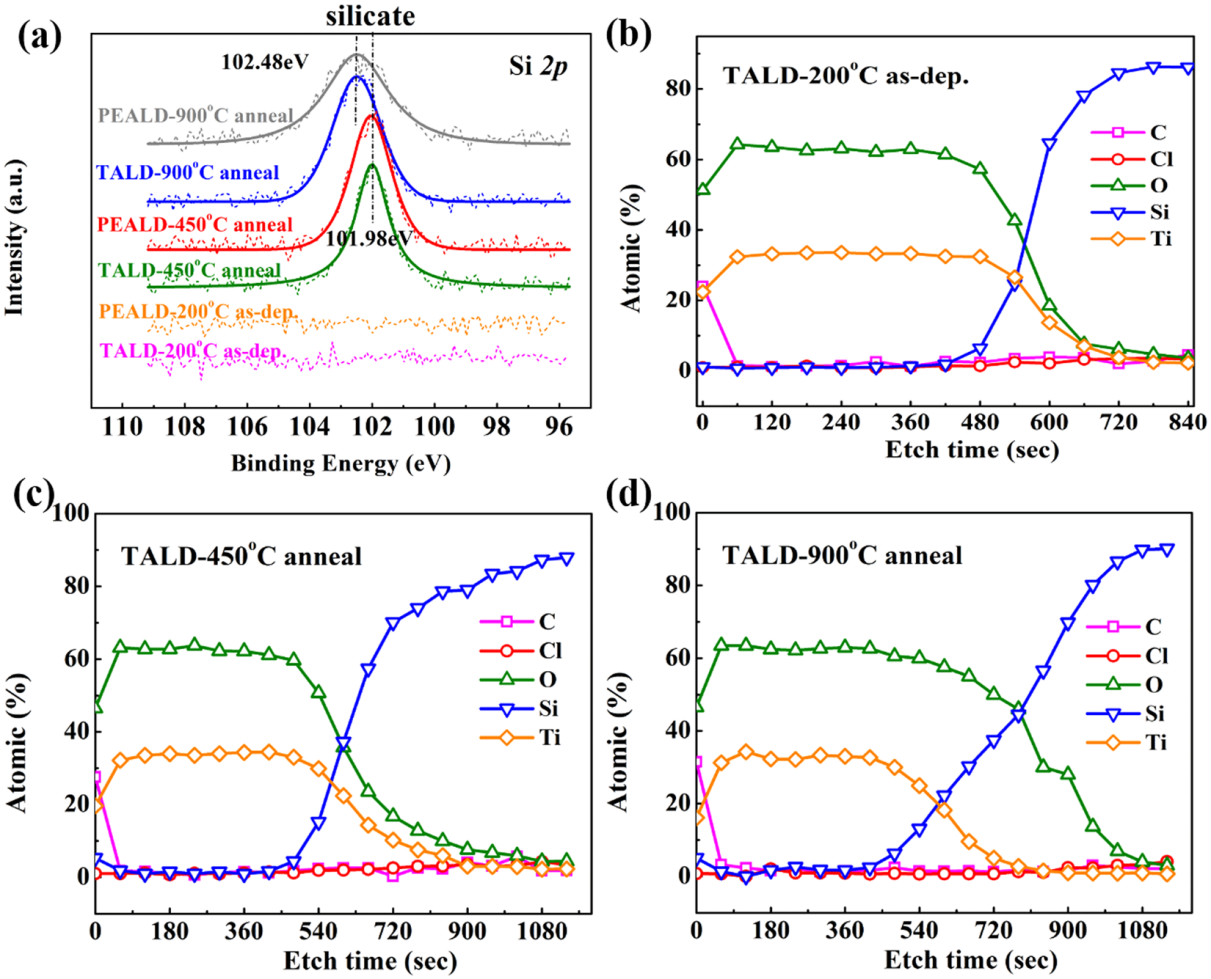
(**a**) Si 2p narrow-scan XPS spectra of as-deposited at 200 °C, 450 °C and 900 °C annealed TALD and PEALD TiO_2_ films surface on Si. XPS depth profiles for (**b**) as-deposited at 200 °C, (**c**) 450 °C and (**d**) 900 °C annealed TALD-TiO_2_ films on Si using Ar^+^ ions etching.

After 600 °C anneal, there is no obvious change in XPS spectra of ZrO_2_ and HfO_2_ films on Si. However, it is found that after 450 °C and 900 °C thermal treatment, the Si 2p signal appears on the surface of TiO_2_ films/Si. Figure 2(a) records Si 2p narrow-scan XPS spectra of as-deposited, 450 °C and 900 °C annealed TALD- and PEALD-TiO_2_ films surface. No Si 2p signal is detected on the surface of as-deposited TALD and PEALD samples. After 450 °C anneal, both samples exhibit Si 2p peaks at 101.98 eV, assigned to the chemical bond of Ti-O-Si. When further raising the anneal temperature to 900 °C, the Si 2p peaks shift to higher binding energy of 102.48 eV, indicating that more Si from substrate diffuses onto TiO_2_ film surface and produce more Ti-silicate^23,24^.

The XPS composition data attest that the atomic ratio of Si: Ti in the film surface is 0.25:1.00 at 450 °C and 0.31:1.00 at 900 °C for TALD samples, and in PEALD samples surface, the atomic ratio of Si: Ti is 0.28: 1.00 at 450 °C and 0.55:1.00 at 900 °C, respectively. The content of Si element on the surface of annealed TiO_2_ films is rather high, so XPS depth analysis on as-deposited, 450 °C and 900 °C annealed TALD TiO_2_ films was carried out by 1000 eV Ar^+^ ions sputtering so as to clarify the source of Si, as indicated in Fig. 2(b–d). Atomic percent content of different elements was recorded every 60 s and the etch rate is around 0.56 Å/s. There is hardly Si signal for as-deposited TiO_2_ film surface, but ~5 at% Si can be clearly observed on the annealed TiO_2_ film surface. After the first etching process, Si content decreases to zero sharply with increased Ti and O contents, whose ratio is among 1.8~2.1, in basic agreement with the stoichiometric TiO_2_. When it comes to 420 s, Si starts to emerge and increase gradually, and Ti and O contents become low. The interfacial diffusion layer of (TiO_2_)_m_(SiO_2_)_n_ is formed between TiO_2_ films and Si substrate because TiO_2_ film is thermodynamically unstable on Si substrate, which easily diffuses into the film and reacts with TiO_2_ during thermal treatment, resulting in formation of Ti-silicate^25,26^. In addition, with the increase of anneal temperature, the thickness of interfacial diffusion layer becomes larger, which can be verified by the following thickness measurement.

**Figure 3:**
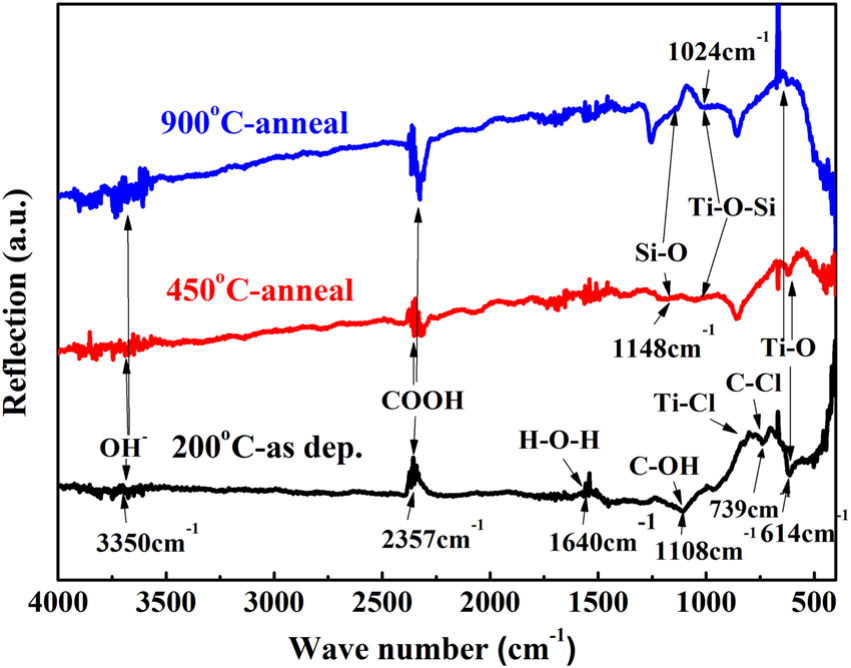
FTIR spectra of as-deposited at 200°, 450 °C and 900 °C annealed TALD TiO_2_ films.

FTIR spectra were also utilized to obtain the chemical group information of TALD-samples before and after thermal anneal, as recorded in Fig. 3. As-deposited sample show Ti-O bond at 614 cm^−1^ with Ti-Cl bond at 811 cm^−1^, C-Cl bond at 739 cm^−1^ and C-OH bond at 1108 cm^−1^, indicating the chlorine residue in film from TiCl_4_ precursor and surface organic carbon contamination due to the lower deposition temperature of 200 °C. Besides Ti-O bond, 450 °C- and 900 °C-annealed TiO_2_ films still display Ti-O-Si and Si-O modes of 1024 cm^−1^ and 1148 cm^−1^ with the disappearance of the Ti-Cl, C-Cl and C-OH bonds, confirming the Si element diffusion into films and removal of Cl residue and organic component after high temperature anneal.

**Figure 4:**
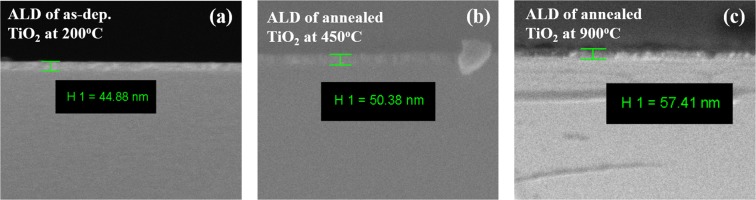
Cross-section FESEM images of TALD TiO_2_ films before chemical test. (**a**) as-deposited at 200 °C. (**b**) 450 °C anneal. (**c**) 900 °C anneal.

SEM and AFM were conducted to characterize the film surface morphologies. Besides, the film thickness can be determined using spectroscopic ellipsometry and cross-section SEM images. The thicknesses of as-deposited and annealed TALD and PEALD TiO_2_, ZrO_2_ and HfO_2_ films from spectroscopic ellipsometry before chemical test are summarized in Table 1. Evidently ZrO_2_ and HfO_2_ films become thinner after anneal, which can be ascribed to remove of oxygen vacancy and densification of films. However, TiO_2_ films become thicker after thermal treatment, especially for 900 °C samples with remarkable thickness increase. This result is also verified by cross-section FESEM images of TALD TiO_2_ films on Si substrate, as seen in Fig. 4.

**Table 1:** Thicknesses of as-deposited and annealed TiO_2_, ZrO_2_ and HfO_2_ films prepared by TALD and PEALD from spectroscopic ellipsometry.

TiO_2_ (1000 cycles)	TALD			PEALD		
	as dep.	post-anneal		as dep.	post-anneal	
	200 °C	450 °C	900 °C	200 °C	450 °C	900 °C
	44.0 nm	49.6 nm	57.9 nm	17.5 nm	18.1 nm	33.8 nm
**ZrO**_**2**_ (**500 cycles**)	**TALD**			**PEALD**		
	250 °C-as dep.		600 °C-anneal	250 °C-as dep.		600 °C-anneal
	38.1 nm		34.8 nm	40.1 nm		37.5 nm
**HfO**_**2**_ (**300 cycles**)	**TALD**			**PEALD**		
	250 °C-as dep.		600 °C-anneal	250 °C-as dep.		600 °C-anneal
	37.9 nm		35.4 nm	35.8 nm		32.2 nm

**Figure 5:**
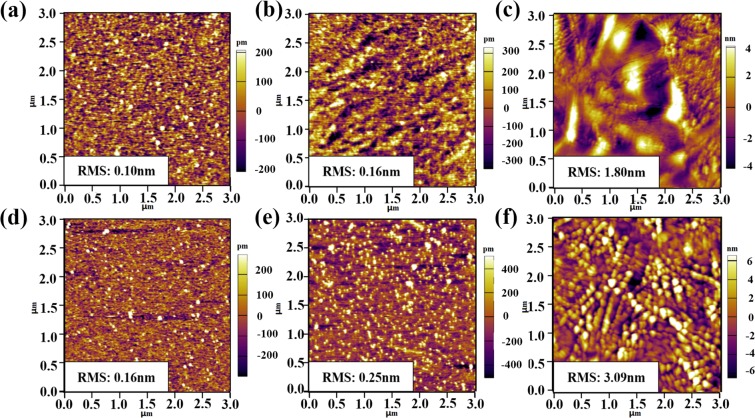
AFM images of as-deposited and annealed TALD and PEALD TiO_2_ films. TALD: (**a**) as-deposited at 200 °C, (**b**) 450 °C anneal and (**c**) 900 °C anneal; PEALD: (**d**) as-deposited at 200 °C, (**e**) 450 °C anneal and (**f**) 900 °C anneal.

Figure 5 is AFM images of as-deposited and annealed TALD and PEALD TiO_2_ films. Thanks to the advantage of ALD technique, large area uniformity, the 200 °C as-deposited and 450 °C annealed films are of little roughness with root-mean-square (RMS) value of 0.2 nm, especially for samples formed by TALD. It can be seen from Fig. 5(c,f), after 3-hour 900 °C post annealing in N_2_ atmosphere, surface morphology of TALD- and PEALD-TiO_2_ films have greatly changed with small particles growing and gathering, the surface roughness increased to 1.8 nm and 3.1 nm respectively. Normally, thermal treatment at high temperature can enlarge grain size in the films, resulting in better crystallinity. Further discussion on structure of samples will be done according to XRD and XPS data.

**Figure 6:**
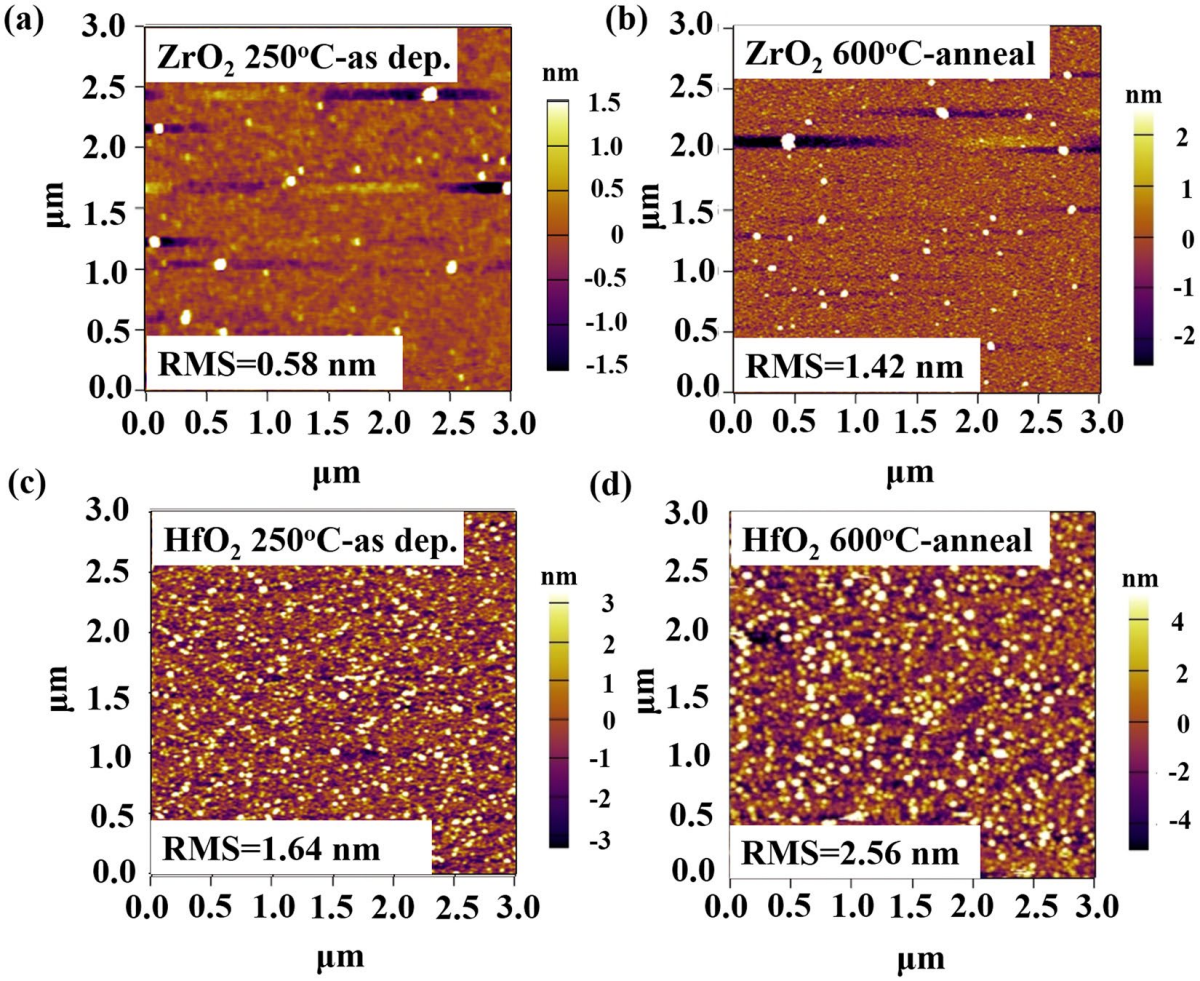
AFM images of as-deposited and annealed TALD ZrO_2_ and HfO_2_ films. (**a**,**c**) As-deposited at 250 °C; (**b**,**d**) 600 °C anneal.

**Figure 7:**
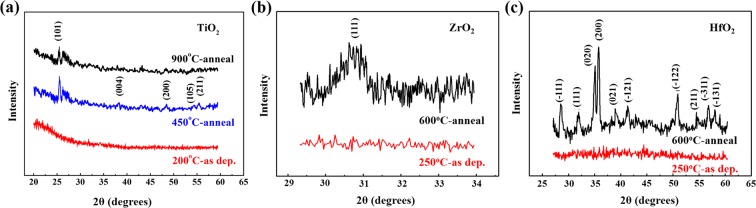
GIXRD patterns of as-deposited and annealed TALD TiO_2_, ZrO_2_ and HfO_2_ ultrathin films.

Similarly, AFM images of as-deposited and annealed TALD ZrO_2_ and HfO_2_ films are presented in Fig. 6. After 600 °C annealing, the ZrO_2_ films have relatively flat surfaces (RMS: 1.42 nm) without easily recognized grains, indicating poor crystallinity. The GIXRD pattern in Fig. 7(b) also proves this. Whereas 600 °C annealed HfO_2_ films contain quite a few 20~30 nm particles with enhanced RMS of 2.56 nm. The XRD result in Fig. 7(c) reveals that stable HfO_2_ monoclinic phase has been formed.

Due to thinner thickness of metal oxide films deposited by ALD technique, GIXRD with the help of synchrotron radiation was introduced to perform phase analysis. Figure 7 illustrates the GIXRD patterns of as-deposited and annealed TALD TiO_2,_ ZrO_2_ and HfO_2_ ultrathin films. As-deposited TiO_2_ samples at 200 °C in Fig. 7(a) are amorphous without any diffraction peaks. 450 °C annealed samples show relatively stronger (101) peak at 25.2° and weak peaks of (004), (200), (105) and (211), which can be assigned to the anatase phase. In addition, a wide peak at 27.5° corresponds to the slight rutile phase. So the mixture of main anatase and slight rutile phases exists in 450 °C TiO_2_ films. Nevertheless, after 900 °C anneal, the TiO_2_ film crystallinity becomes worse, as confirmed by the reduced (101) peak intensity and some disappeared weak peaks. It has been reported that TiO_2_ forms the anatase phase when thermal-treated between 300 and 500 °C, and then turns to the rutile structure above 800 °C anneal^27^. Herein the evident phase discrepancy can be attributed to the severe Si diffusion between TiO_2_ ultrathin films and Si substrate, leading to the interfacial diffusion layer and the poor crystalline of TiO_2_. Meanwhile, Si-doped TiO_2_ films also prevent the transform from anatase to rutile phase. The XRD results are in good agreement with the mentioned-above XPS and FTIR analyses.

Comparing GIXRD patterns of ZrO_2_ and HfO_2_ films after 10-minute 600 °C RTA (Fig. 7(b,c)), obviously HfO_2_ films have changed from amorphous to monoclinic phase with sharp peaks. The calculated grain size is 23.3 nm by Schereer equation, in consistent with AFM observation. While under the same anneal condition, the crystallinity of ZrO_2_ films is not as good as that of HfO_2_ and a weak and broad diffusion peak at 30.5° implies the trend of crystallinity.

### Chemical stability in chemical liquid media

**Figure 8:**
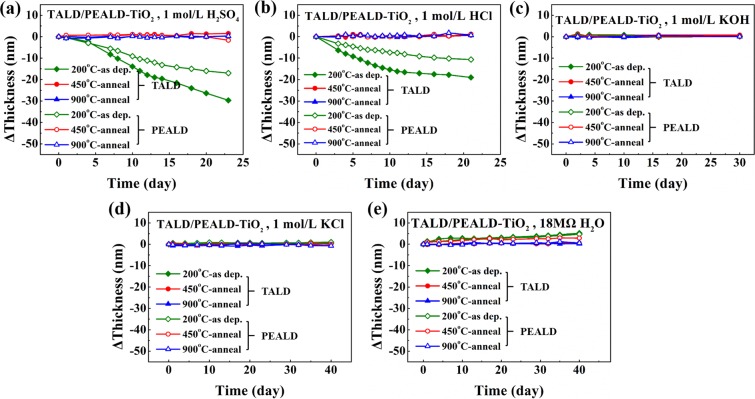
The thickness variation vs. immersion time for all as-deposited and post-annealed TALD/PEALD TiO_2_ films in various chemical liquid media: (**a**) 1 mol/L H_2_SO_4_, (**b**) 1 mol/L HCl, (**c**) 1 mol/L KOH, (**d**) 1 mol/L KCl and (**e**) 18 MΩ water.

To study anti-corrosion property of group IV metal oxides, we immersed a subset of as-deposited and annealed TALD and PEALD samples in various chemical liquid media with a range of pH value, including acid solutions (1 mol/L H_2_SO_4_, 1 mol/L HCl), an alkaline solution (1 mol/L KOH) and neutral solutions (1 mol/L KCl, 18MΩ pure water). Film thickness was examined using spectroscopic ellipsometry after regular intervals. Figure 8 illustrates the thickness change dependence on immersion time for all as-deposited and post-annealed TALD/PEALD-TiO_2_ films in various chemical environments, so as to evaluate the influence of anneal and deposition method on etching resistance of TiO_2_ films.

From the point of view of film thickness variation, TiO_2_ thin films in alkaline and neutral solutions of 1 mol/L KOH and 1 mol/L KCl are very stable, and the film thickness has hardly changed, even if as-deposited ones at 200 °C without post-anneal, as indicated in Fig. 8(c,d). In Fig. 8(e), as-deposited and 450 °C annealed TiO_2_ samples in 18 MΩ water show a little slight thickness increase due to the hydration role; after 900 °C post-anneal, the film thickness basically keeps constant.

However, as-deposited TiO_2_ thin films are unstable in acidic solutions with slow dissolution during the immersion duration, resulting in the visual change of the films in reflection color. The etch rates in 1 mol/L H_2_SO_4_ and 1 mol/L HCl in Fig. 8(a,b) are summarized in Table 2.

**Table 2:** Etch rates of TALD- and PEALD-TiO_2_ films in 1 mol/L H_2_SO_4_ and 1 mol/L HCl.

Solution	TALD-TiO_2_			PEALD-TiO_2_		
	as dep.	post-anneal		as dep.	post-anneal	
	200 °C	450 °C	900 °C	200 °C	450 °C	900 °C
H_2_SO_4_	1.39 nm/day	—	—	0.81 nm/day	—	—
HCl	0.82 nm/day	—	—	0.48 nm/day	—	—

Evidently H_2_SO_4_ aqueous solution is more corrosive to as-deposited TiO_2_ films than HCl solution at the same molar concentration, because sulfuric acid is a binary strong acid, which can ionize twice amount of H^+^ of single hydrochloric acid. Moreover, the etch rate of as-deposited PEALD samples in acid drops by about 41% than that of as-deposited TALD ones, which can be ascribed to the fact that PEALD-derived samples have enhanced film density and lower impurity concentration due to the O_2_ plasma’s higher surface reactivity than only thermal energy-assisted process alone^28^.

After annealed 450 °C and 900 °C for 3 h, the chemical stability of TALD and PEALD TiO_2_ ultrathin films on Si is improved significantly with nearly unchanged thickness in strong acidic solutions over test period of 20 days in Fig. 8(a,b). As described above in Figs 2 and 7(a), the XPS and XRD results confirm that the longer time annealing at 450 °C and 900 °C for 3 h causes the Si diffusion into TiO_2_ films to form the amorphous Ti-silicate containing partial crystalline anatase TiO_2_ main phase. The amorphous Ti-silicate and crystalline TiO_2_ exhibit better anti-acid corrosion performance than amorphous TiO_2_. Especially, the formation of 5% Si-doped TiO_2_ ultrathin layers on sample surfaces in Fig. 2(c,d) also plays an important role in enhancing the acid-endurance.

**Figure 9:**
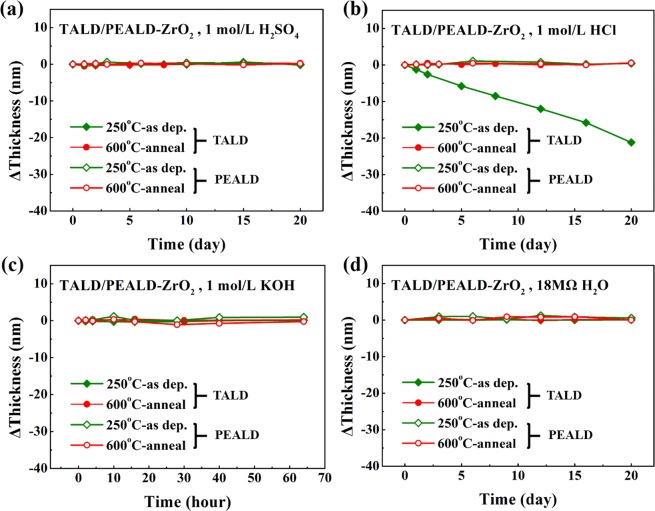
The thickness variation vs. time for all 250 °C as-deposited, 600 °C-annealed TALD/PEALD-ZrO_2_ films in various chemical liquid media: (**a**) 1 mol/L H_2_SO_4_, (**b**) 1 mol/L HCl, (**c**) 1 mol/L KOH and (**d**) 18 MΩ water.

The thickness variation dependence on immersion time for all as-deposited and post-annealed TALD/PEALD-ZrO_2_ films in various chemical environments are recorded in Fig. 9. Except for as-deposited TALD ZrO_2_ films dissolving in 1 mol/L HCl with the etch rate of 1.06 nm/day, all other ZrO_2_ samples exhibit better stability in different aqueous environments during the whole 20-day test, including annealed TALD and all PEALD samples. Herein, the influence of PEALD is much more significant without any etching than TiO_2_ case for O_2_ plasma contains more reactive radical and ionic species, beneficial to the corrosion-endurance ZrO_2_ films’ growth. Meanwhile annealing at high temperature may remove the residual impurities in films, leading to densification and crystallization trend of ZrO_2_ films. This produces positive effects on improving anti-corrosion property of ZrO_2_ films, as indicated in Fig. 9(b).

**Figure 10:**
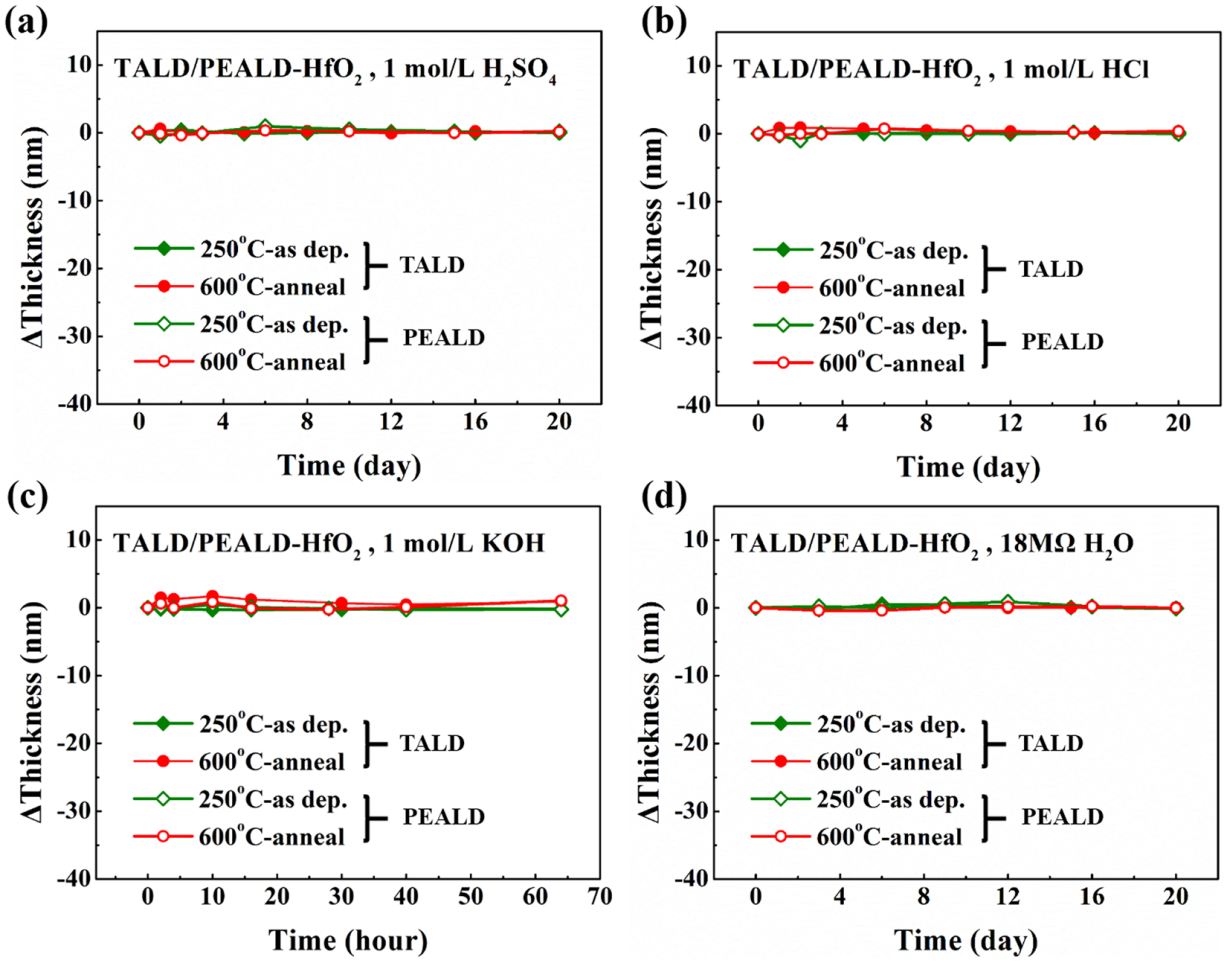
The thickness variation vs. time for all 250 °C as-deposited, 600 °C-annealed TALD/PEALD-HfO_2_ films in various chemical liquid media: (**a**) 1 mol/L H_2_SO_4_, (**b**) 1 mol/L HCl, (**c**) 1 mol/L KOH and (**d**) 18 MΩ water.

Figure 10 displays the anti-etching characteristics of all TALD and PEALD HfO_2_ films in various chemical liquid media. Among three kinds of group IV metal oxides, HfO_2_ films may be the best candidate as barrier layer in caustic environments, because after the long-time immersion in any acidic, alkaline and neutral solutions, all the HfO_2_ samples show negligible change in thickness, no matter from TALD and PEALD or before and after anneal. So, we chose HfO_2_ films as one candidate to further examine the corrosion resistance by electrochemical measurements.

### Electrochemical result

We deposited 300 cycles HfO_2_ films (~36 nm-thick) onto 304 stainless steels (304 SS) by TALD and PEALD. It was reported that the anti-corrosion properties of ALD Al_2_O_3_ thin films on steel could be improved by the H_2_–Ar plasma pre-treatment^29^. In our experiment, 304 SS was treated by *in-situ* 50 s H_2_-Ar plasma pulse before HfO_2_ deposition so as to study the effect of the H_2_ pre-treatment.

**Figure 11:**
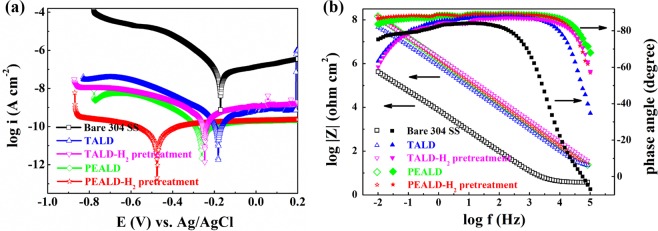
(**a**) Polarization curves and (**b**) Bode plots of untreated bare 304 SS and TALD- and PEALD-HfO_2_ coated 304 SS with and without H_2_ plasma pretreatments.

The polarization curves and Bode plots for the EIS data measured at OCP for the bare and coated 304 SS in 1 mol/L KCl are shown in Fig. 11. Only a peak at the corrosion potential can be observed in the polarization curves of each sample, indicating the HfO_2_ coating films are electrochemically inert^30^. Compared with the bare 304 SS, the corrosion potential (E_corr_) of TALD- and PEALD-HfO_2_ coated 304 SS with and without H_2_ plasma pretreatments, shifts to more negative potential, leading to improved corrosion resistance. Corrosion current density (I_corr_) is another key factor to evaluate the corrosion reaction kinetics, which is usually proportional to the corrosion rate and can be obtained by Tafel fit analysis^16,31^. Additionally, polarization resistance (R_p_) and porosity (P) are calculated and presented in Table 3, using Eqs 1 and 2.

**Table 3:** Parameters related to the polarization curves in Fig. 11(a) for untreated bare 304 SS and TALD- and PEALD-HfO_2_ coated 304 SS with and without H_2_ plasma pretreatments.

	H_2_ plasma pre-treatment	E_corr_ (V)	I_corr_ (A·cm^−2^)	R_p_ (Ω · cm^2^)	P (%)
Bare 304 SS	No	−0.17	1.09E-07	3.49E + 05	100
TALD-HfO_2_	No	−0.18	3.92E-10	2.77E + 07	0.95
	Yes	−0.24	3.55E-10	4.73E + 07	0.24
PEALD-HfO_2_	No	−0.26	1.78E-10	9.29E + 07	0.15
	Yes	−0.48	5.25E-11	3.43E + 08	0.01

The HfO_2_ coatings lead to a marked decrease in corrosion current density (I_corr_) from 1.09 × 10^−7^ A/cm^2^ for bare 304 SS to ~10^−10^ A/cm^2^ with three orders of drop. Accordingly, polarization resistance (R_p_) increases by three orders of magnitude. The porosity (P) represents the nominal uncoated surface fraction of the 304 SS substrate based on the ratio of polarization resistance without and with HfO_2_ coatings. The smaller the porosity (P) is, the better the corrosion resistance is. Among all samples, PEALD-HfO_2_ coated 304 SS with H_2_ plasma pretreatments exhibits the most encouraging anti-corrosion property with most negative potential of −0.48 V, lowest corrosion current density of 5.25 × 10^−11^ A/cm^2^, largest polarization resistance of 3.43 × 10^8^ Ω · cm^2^ and smallest porosity of 0.01. This can be ascribed to the combination role of the H_2_ plasma pre-treatment and the high O_2_ plasma activity during PEALD. The removal of organic contamination and improvement of adhesion to the 304 SS caused by the H_2_ plasma pre-treatment are beneficial to anti-corrosion property^29^.

Electrochemical impedance spectra (Bode plots) of different samples are presented in Fig. 11(b). The HfO_2_ coating properties are evaluated from low frequency to high frequency. Compared to the bare 304 SS, all HfO_2_ coated 304 SS samples show a clear increase in the global impedance. Moreover, PEALD HfO_2_ coated 304 SS samples maintain a higher phase angle in a wide frequency range from 10^−2^ Hz to 10^5^ Hz, giving better corrosion protection. Above all, the electrochemical results obtained from the LSV and the EIS confirm that PEALD HfO_2_ coating onto H_2_ plasma pretreated 304 SS has optimal corrosion-endurance.

## Conclusion

TiO_2_, ZrO_2_ and HfO_2_ thin films were deposited on p-type Si (100) with nature oxide layer by the technique of TALD and PEALD. A variety of chemical liquid media including 1 mol/L H_2_SO_4_, 1 mol/L HCl, 1 mol/L KOH, 1 mol/L KCl, and 18 MΩ deionized water were used to test and compare chemical stability of all these as-deposited group IV metal oxides thin films, as well as post-annealed samples. In acidic solutions, TALD/PEALD HfO_2_ films, whether annealed or not, exhibit the best chemical stability without any change in thickness after long time immersion. Except as-deposited TALD-ZrO_2_ films with slowly etch rate of 1.06 nm/day in 1 mol/L HCl, all other ZrO_2_ films show better anti-acid stability, indicating the role of introduction of plasma O_2_ in PEALD and post-thermal treatment. As-deposited TiO_2_ films are relatively unstable in acidic solutions of H_2_SO_4_ and HCl, where the etch rate is 1.39 nm/day and 0.82 nm/day for TALD ones, and 0.81 nm/day and 0.48 nm/day for PEALD ones, respectively. The etch rate of PEALD samples in acid solutions drops by about 41%, compared to TALD ones. After post-annealing, TiO_2_ films have good corrosion endurance, which is ascribed to the crystallization transition from amorphous to anatase main phase and the formation of 5% Si-doped TiO_2_ ultrathin layers on sample surfaces. In basic and neutral solutions, HfO_2_, ZrO_2_, and TiO_2_ all show excellent corrosion resistance property with negligible change in thickness. Simultaneously, compared with bare 304 SS, 304 SS with HfO_2_ coating shows enhanced anti-corrosion property, and 36 nm PEALD-HfO_2_ is found to have a lower corrosion rate than TALD-HfO_2_ ones, which is ascribed to the improved film quality when activated O_2_ plasma is used. The pre-treatment of plasma H_2_ to substrate of SS can reduce interfacial impurities and porosity of overlayers with increased corrosion resistance characteristics. Electrochemical analysis yields an exponential decay of the corrosion current density by four orders of magnitude. Above all, using PEALD technique, post-annealing process and plasma H_2_ pre-treatment can improve the chemical stability and corrosion resistance of group IV metal oxide coatings. As a result, HfO_2_, ZrO_2_, and TiO_2_ ultrathin films derived from TALD and PEALD allows various protective applications in several commonly-used chemical liquid environments.

## Materials and Methods

### Preparation of TiO_2_, ZrO_2_ and HfO_2_ films by TALD and PEALD

TiO_2_ films were deposited at 200 °C, ZrO_2_ and HfO_2_ films were deposited at 250 °C, in a commercial Picosun SUNALE™ R-200 advanced PEALD system, which can be operated under PEALD and TALD modes. P-type Si (100) substrates (1 ~ 10 Ω · cm) were ultrasonically cleaned by acetone, alcohol and deionized water in turn without removing native oxide. TiCl_4_, Zr[N(C_2_H_5_)CH_3_]_4_ (TEMAZ) and Hf[N(C_2_H_5_)CH_3_]_4_ (TEMAH) as metal precursors were used at room temperature, 150 °C and 155 °C, respectively. H_2_O and O_2_ plasma were adopted as oxygen source for deposition of metal oxide by TALD and PEALD, respectively, where remote plasma power and O_2_ gas flow rate were 2,500 W and 160 sccm. For TALD-derived metal oxide thin films, all precursors’ pulse time was 0.1 s, followed by a 4 s N_2_ purge step to remove extra precursors and by-products. For PEALD-derived samples, the pulse and purge time of metal precursor kept unchanged, but pulse and purge time of plasma O_2_ was extended to 13.5 s and 10 s, respectively.

All as-deposited samples were also annealed at high temperature to evaluate the effect of thermal treatment on chemical stability. Both TALD- and PEALD-TiO_2_ films were heated in tube furnace at 450 °C and 900 °C for 3 h under N_2_ atmosphere, while annealed ZrO_2_ and HfO_2_ films were made in O_2_ environment at 600 °C for 10 min by rapid thermal annealing (RTA) to promote crystallization.

As-deposited and post-annealed samples were immersed in various chemical liquid media for various time, including 1 mol/L H_2_SO_4_, 1 mol/L HCl, 1 mol/L KOH, 1 mol/L KCl, and 18 MΩ deionized water. All the samples were stored in acid/alkaline-resistant boxes at room temperature in dark while control samples were placed in air.

### Characterization

The physical thickness of films (before and after immersion) was estimated by spectroscopic ellipsometry (Sopra GES-5) at an incidence angle of 75° and with wavelengths between 300 nm to 800 nm at increments of 10 nm. The data were then fit using the Cauchy (epsi) model for TiO_2_, ZrO_2_ and HfO_2_ films to acquire thickness value. A fixed native SiO_2_ layer (1~2 nm) was considered in the model at the Si-metal oxide interface using the software of WinElli. X-ray photoelectron spectroscopy (XPS, Thermo Fisher K-Alpha, USA) was explored to characterize the chemical state and component of samples, using a monochromatic Al *Kα* source (hν = 1,486.6 eV) for excitation of photoelectrons and binding energy scale was calibrated using C 1 s peak at 284.6 eV. Fourier transform infrared spectroscopy (FTIR) was used to obtain the chemical group information of the thin films using a pristine Si substrate as a reference. Crystallinity and phase structures of the films were analyzed by grazing incidence X-ray diffraction (GIXRD, Shanghai Synchrotron Radiation Facility, BL14B1) system with the light source energy of 10k eV and grazing incidence of 0.12° due to the extremely thin thickness of the films. The surface topography was recorded by atomic force microscopy (AFM, Cypher, USA) and field emission scanning electron microscopy (FESEM, Zeiss Ultra 55, German). The cross-section FESEM image was also recorded to corroborate the thickness of samples.

**Figure 12:**
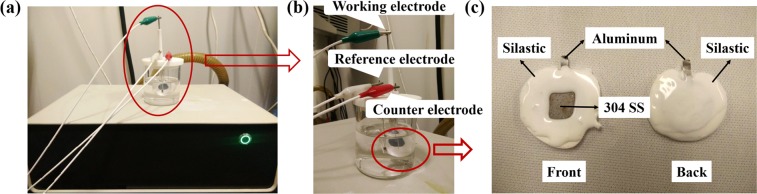
Experimental setup for electrochemical measurements including (**a**) electrochemical workstation, (**b**) three-electrode test and (**c**) sample package picture.

Electrochemical measurements were performed by CHI660E electrochemical workstation and three-electrode tests where bare or coated 304 SS was used as the working electrode, a platinum wire as the counter electrode, saturated Ag/AgCl solution as a reference, and 1 mol/L KCl as the electrolyte and corrosion solution. 704 silicone rubber was used to encapsulate the surface of the sample. The reverse side of the sample was sealed after the extraction of the Al electrode ears equipped with the electrochemical workstation. Only a square area of about 1 × 1 cm^2^ was left on the front for testing. Figure 12 shows the experimental setup for electrochemical measurements including electrochemical workstation, three-electrode test and sample package picture.

During measurement, the open circuit potential (OCP) was detected for one hour to attain a steady potential, then the electrochemical impedance spectroscopy (EIS) measurements were conducted in a frequency range of 10^−2^ to 10^5^ Hz at OCP. The linear sweep voltammetry (LSV) data were collected at a rate of 20 mV/min and the corrosion potentials, corrosion currents, polarization resistances and porosities were calculated based on the polarization curves using the Stern-Geary equation^30^

1 $${R}_{P}=\frac{{b}_{a}{b}_{c}}{{i}_{corr}\,\mathrm{ln}\,(10)({b}_{a}+{b}_{c})}$$

where b_a_ and b_c_ are the slopes of the anodic and cathodic branches of the Tafel plot, respectively. The electrochemical porosity of the films deposited onto SS was obtained by comparing R_p_ of coated and bare SS^29^.

2 $$P=\frac{{R}_{p-bare}}{{R}_{p-ALD}}\times 100 \% \,$$
